# NHGNN-DTA: a node-adaptive hybrid graph neural network for interpretable drug–target binding affinity prediction

**DOI:** 10.1093/bioinformatics/btad355

**Published:** 2023-05-30

**Authors:** Haohuai He, Guanxing Chen, Calvin Yu-Chian Chen

**Affiliations:** Artificial Intelligence Medical Research Center, School of Intelligent Systems Engineering, Shenzhen Campus of Sun Yat-sen University, Shenzhen, Guangdong 518107, P.R. China; Artificial Intelligence Medical Research Center, School of Intelligent Systems Engineering, Shenzhen Campus of Sun Yat-sen University, Shenzhen, Guangdong 518107, P.R. China; Artificial Intelligence Medical Research Center, School of Intelligent Systems Engineering, Shenzhen Campus of Sun Yat-sen University, Shenzhen, Guangdong 518107, P.R. China; Department of Medical Research, China Medical University Hospital, Taichung 40447, Taiwan; Department of Bioinformatics and Medical Engineering, Asia University, Taichung 41354, Taiwan

## Abstract

**Motivation:**

Large-scale prediction of drug–target affinity (DTA) plays an important role in drug discovery. In recent years, machine learning algorithms have made great progress in DTA prediction by utilizing sequence or structural information of both drugs and proteins. However, sequence-based algorithms ignore the structural information of molecules and proteins, while graph-based algorithms are insufficient in feature extraction and information interaction.

**Results:**

In this article, we propose NHGNN-DTA, a node-adaptive hybrid neural network for interpretable DTA prediction. It can adaptively acquire feature representations of drugs and proteins and allow information to interact at the graph level, effectively combining the advantages of both sequence-based and graph-based approaches. Experimental results have shown that NHGNN-DTA achieved new state-of-the-art performance. It achieved the mean squared error (MSE) of 0.196 on the Davis dataset (below 0.2 for the first time) and 0.124 on the KIBA dataset (3% improvement). Meanwhile, in the case of cold start scenario, NHGNN-DTA proved to be more robust and more effective with unseen inputs than baseline methods. Furthermore, the multi-head self-attention mechanism endows the model with interpretability, providing new exploratory insights for drug discovery. The case study on Omicron variants of SARS-CoV-2 illustrates the efficient utilization of drug repurposing in COVID-19.

**Availability and implementation:**

The source code and data are available at https://github.com/hehh77/NHGNN-DTA.

## 1 Introduction

Drug discovery is a time consummation and tremendous economic cost task (Dickson and Gagnon 2004). It is usually carried out through high-throughput screening *in vitro* experiments. However, those methods consume a lot of money and are constrained by human abilities, making it impossible to screen completely from large-scale drug databases (Li *et al.* 2022). Identifying novel drug–target affinity (DTA) is a crucial stage for drug discovery (Zheng *et al.* 2020). The DTA prediction based on machine learning (ML) can accelerate the process of drug discovery and reduce money consumption (Schneider 2018, Dara *et al.* 2022).

Before ML-based methods, DTA prediction methods were based on molecular docking and molecular dynamics simulations (Singh *et al.* 2022, Du *et al.* 2023). These methods rely on high-precision 3D structures of molecules and proteins (Yang *et al.* 2022), so they are time-consuming or even unusable when the 3D structures of proteins are unknown (Dhakal *et al.* 2022).

The ML model predicts the interaction activity score using only the text information of drugs and proteins, reducing the cost of drug discovery, expanding the search space, and avoiding missing potential candidates through in silico prediction. It aims to predict the interaction activity score *in silico* and only uses the text information of drugs and proteins. Small drug molecules are usually characterized by a simplified molecular input line entry system (SMILES) (Weininger 1988), which is a specification that explicitly describes the molecular structure with ASCII strings. The DTA reflects the degree of interaction between drug–protein (DP) pairs, which is usually expressed by dissociation constant ($K_{d}$), inhibition constant ($K_{i}$), or half maximum inhibition concentration ($IC_{50}$). $IC_{50}$ depends on the concentration of the target and ligand. A low $IC_{50}$ value indicates strong binding, while the $pIC_{50}$ is the negative logarithm of $IC_{50}$. Although there are already deep learning frameworks that can predict protein structures with high accuracy (Jumper *et al.* 2021, Baek *et al.* 2021). Meanwhile, researchers are also trying to use 3D structures for drug discovery subtasks, such as molecular properties (Liu *et al.* 2022) and drug–drug interaction prediction (He *et al.* 2022). However, they still consume a lot of computational resources and fail to effectively capture the key information required for DTA prediction: the position of DP interaction pockets and connected edges. Therefore, ML-based DTA predictions still do not use the 3D structure of drugs and proteins.

Currently, ML-based DTA predictions are mainly divided into two schemes: sequence-based and graph-based methods. Sequence-based methods take text embeddings of small drug molecules and proteins as output and use convolutional neural networks to predict DTA. However, sequence-based DTA prediction methods only take one-dimensional sequence information as input, ignoring the structural features of drugs and proteins. To solve this problem, graph-based methods have gradually captured scholars’ attention. GraphDTA (Nguyen *et al.* 2021a) pioneered the introduction of Graph Neural Networks (GNNs) into DTA prediction, where drug molecular graphs were used as drug representations. However, they still treated proteins as one-dimensional sequences. Then, to make full use of the structural information of proteins, DrugVQA (Zheng *et al.* 2020) constructs a protein graph using the contact map, and treats amino acids as nodes of the protein graph. However, current graph-based approaches treat drugs and proteins as separate graphs, where their features are usually only extracted by GNNs. It is worth noting that the node features in the drug and protein graphs are only artificially selected for some atomic and amino acid properties, thus making the DTA model lack generalization.

**Figure 1.**
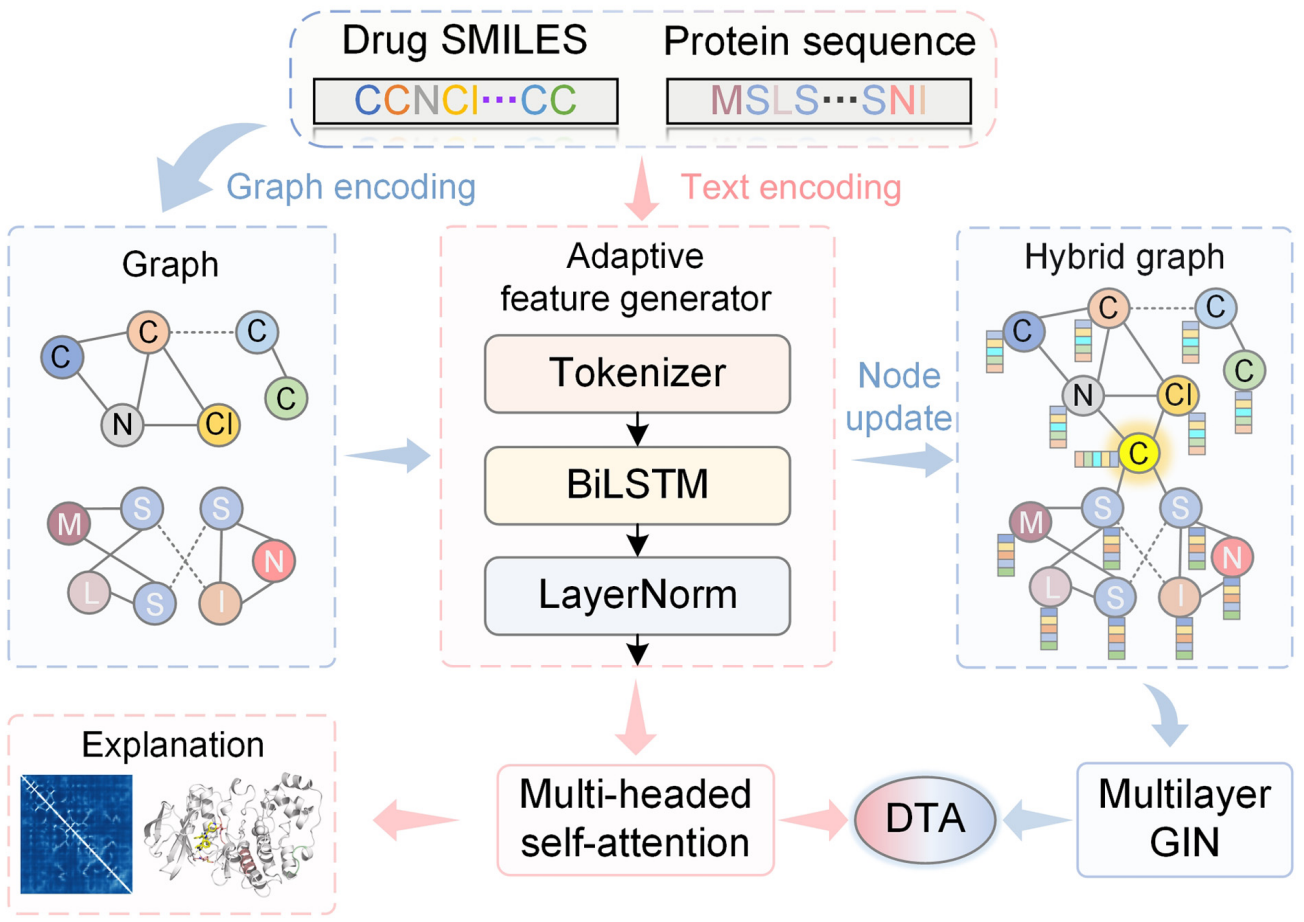
The flowchart of NHGNN-DTA for drug design. First, the graphs of drugs and proteins and the features of drug atoms and protein amino acids are obtained through graph and text encoding. The text embeddings are fed into the adaptive feature generator, processed through the tokenizer, BiLSTM and LayerNorm layers, and fed into the multi-head self-attention layer, which outputs sequence-based DTA predictions and interpretability. During this process, the feature generator adaptively obtains normalized node feature updates. We combine the graphs of drugs and proteins into a hybrid graph with a unique central node, and blend in adaptive node features, which are then fed into a multilayer GIN to obtain graph-based DTA predictions. Finally, the sequence-based and graph-based prediction results are integrated to obtain the final DTA prediction.

To address this problem, we proposed NHGNN-DTA, a node-adaptive hybrid graph neural network (HGNN) for interpretable DTA prediction. It includes a sequence-based adaptive feature generator and a graph-based HGNN that mixes drug and protein graphs. As shown in Fig. 1, NHGNN-DTA generates node features for drugs and proteins, improving generalization by avoiding manual feature selection. We construct HGNN to combine drug and protein graphs, achieving graph-level interaction. The feature generator obtains atomic and amino acid node features, and we use a central node and LayerNorm layer to address feature drift. Specifically, the feature generator obtains the latent features of atomic nodes in the drug graph and amino acid nodes in the protein graph, where we constructed a well-designed atomic tokenizer and central node to form the HGNN. To solve the feature drift problem, we have adopted LayerNorm layer and interval update method to ensure training convergence and feature normalization. In summary, the model combines the advantages of sequence-based and graph-based methods to not only adaptively obtain drug and protein characterization, but also fully utilize the structural information of drugs and proteins.

Through extensive comparative experiments on two well-known benchmark datasets, Davis (Davis *et al.* 2011) and KIBA (Tang *et al.* 2014), NHGNN-DTA achieved a new state-of-the-art (SOTA) performance in DTA prediction. Under the more realistic three cold-start divisions, NHGNN showed superiority to the SOTA model for all metrics in both datasets. Moreover, we have conducted elaborate ablation studies to prove the necessity of each component of NHGNN-DTA. In addition, visualization of drug and protein weights for some examples confirms that NHGNN-DTA successfully captures the proven interactions. The results demonstrate that NHGNN-DTA has strong interpretability and potential to explore unknown DTA.

## 2 Related works

### 2.1 Sequence-based DTA prediction

DeepDTA (Öztürk *et al.* 2018) firstly uses two independent CNN to extract the features of drug SMILES and protein sequences to achieve affinity prediction. MT-DTI (Shin *et al.* 2019) firstly introduces the attention mechanism (Vaswani *et al.* 2017) for drug representation, which improves the DTA prediction performance and explainability. rzMLP (Qiu *et al.* 2021) uses the global feature aggregation as features, and uses gMLP aggregated features and ReZero layers to smooth the training process for learning complex global features. EnsembleDLM (Kao *et al.* 2021) adopts an ensemble learning method to aggregate predictions with multiple sequence information to improve the accuracy of DTA predictions. FusionDTA (Yuan *et al.* 2022) uses BiLSTM (Graves *et al.* 2013) and attention mechanism to process sequence features as language models.

### 2.2 Graph-based DTA prediction

MGraphDTA (Yang *et al.* 2022) utilizes a multi-scale graph neural network to capture the information on drug substructure. GEFA (Nguyen *et al.* 2021b) characterizes the structural information of proteins through contact maps, in which an attention mechanism is also introduced to enable interactions between drugs and protein amino acid nodes. These methods select certain properties of drugs and proteins as node features, such as atom type, implied valence, and atomic number. Compared to sequence-based methods, graph-based methods focus on building the structure of drug and protein graphs and obtaining internal interactions. However, due to the inconsistent properties of proteins and drugs, graph-based methods can neither construct drugs and molecules in a graph, nor realize the DTA information interaction at the graph level.

## 3 Materials and methods

**Figure 2.**
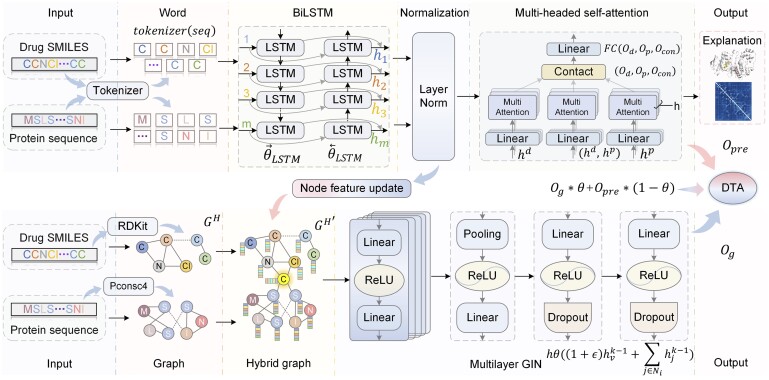
Overview of the NHGNN-DTA framework. The adaptive feature generator is shown in the upper part of the figure. It is designed to generate high-dimensional feature representations of amino acids and atoms. It obtains a good feature representation through a multi-head attention mechanism in the pre-training stage and then dynamically and intermittently transfers the normalized features to the HGNN. As shown in the lower part of the figure, HGNN realizes graph-level information interaction through the only central node “C.” In the joint training of the feature generator and HGNN, the features of the hybrid graph are adaptively obtained for further optimization. Finally, the predictions of the DTA are jointly output.

This study aims to adaptively generate features of drug atoms and protein amino acids and construct graph structures containing atoms and amino acid nodes to predict DTA. To this end, we propose NHGNN-DTA, an ensemble neural network framework for adaptively generating graph node features, which consists of a node feature generator and HGNN. The flowchart of NHGNN-DTA is shown in Fig. 2. The node feature generator encodes the SMILES of drugs and sequences of proteins, and obtains the features of the corresponding nodes. In HGNN, the input graph contains amino acid and drug atom nodes, and the DTA predictions are output through a multilayer graph isomorphism network (GIN) (Xu *et al.* 2019).

### 3.1 Adaptive feature generator

Previous graph-based methods manually set node features based on prior knowledge, which may be limiting. In this study, we use a sequence-based approach to adaptively construct amino acid and atomic node features and apply them to downstream HGNN, which allows for more flexibility. In the sequence-based DTA prediction method, we first encode the SMILES of the drug and the sequence of the protein. To obtain the corresponding embedding for each atom in the drug, we specifically design a tokenizer, which is encoded at the atomic level, and thus ensure the embedding corresponds to an atom or chemical bond. As shown in the upper part of Fig. 2, we use BiLSTM as the main feature generator backbone. It can efficiently extract SMILES and protein sequence features of drugs simultaneously. We use two independent BiLSTMs to extract features from the drug and protein embeddings obtained by the tokenizer as follows: where $h_{i}$ represents the output of BiLSTM, and *text* represents SMILES of drugs and sequences of proteins.


(1) $$(h_{1},h_{2},\ldots ,h_{m})=\text{BiLSTM}(\text{tokenizer}(\text{text})),$$

To obtain an efficient feature representation, we need to extract the features obtained by BiLSTM through a sequence-based method, so we transfer the drug and protein features into a linear attention layer. The computation of linear attention can be described as: where $W\in R^{1*f}$ is the attention weight matrix, while $d_{K}$ is the normalization parameter.


(2) $$\text{LinearAttention}(W,h_{i})=\frac{\;\text{exp}(\frac{W_{h}i}{\sqrt{d_{k}}})}{\sum_{j=1}^{m}e\text{xp}(\frac{W_{h}i}{\sqrt{d_{k}}})},$$

In addition, to enrich the diversity of attention, we use the multi-head attention mechanism as follows:


(3) $$\begin{array}{c} O_{d}=\text{MultiheadAttn}(h^{d}),\\ O_{p}=\text{MultiheadAttn}(h^{p}). \end{array}$$

Then we get the corresponding outputs for drugs and proteins: $O_{d}$ and $O_{p}$. To improve DP pair interactions at the sequence level, we concatenate the outputs of the two linear attention. The concatenated vectors are then further transmitted to the total linear attention layer to realize the information interaction between drugs and proteins. The specific operation can be expressed as:


(4) $$O_{\text{con}}=\text{MultiheadAttn}(\text{Concat}(h^{d},h^{p})).$$

Finally, we feed the fusion output to the fully connected layer to get the DTA predictions $O_{\text{pre}}$ as follows:


(5) $$O_{\text{pre}}=FC(\text{Concat}(O_{D},O_{P},O_{\text{con}})).$$

NHGNN-DTA uses a sequence-based feature generator to extract sequence information of DP pairs, and the performance of the HGNN model depends on the feature extractor’s output. To ensure quality, the feature generator is pre-trained before HGNN training.

### 3.2 Hybrid graph neural network

This study aims to create a hybrid graph with both amino acid and atomic nodes, allowing for interaction between drug and protein graphs. Previous methods ignore DP interactions, but the hybrid graph addresses this issue. A suitable method is needed to construct HGNN for DTA. To this end, we need to find a suitable method to construct HGNN for DTA. Suppose the graph structure of drugs and proteins is expressed as $G\in r^{l*l}$, where *l* is the length of sequence or SMILES, $G_{ij}$ equals 1 when *i* is in contact with *j* node. For the drugs graph, we use RDKit (Landrum *et al.* 2013) to obtain the chemical bond interactions between atoms as the edges of the graph structure to construct the drug molecular graph $G^{d}$. To construct the protein graph, we use pconsc4 (Michel *et al.* 2019) to generate the distance matrix between the residues in the protein. Then, by setting a distance threshold, two residues with a distance smaller than this threshold are regarded as contacts to obtain a contact map, which is regarded as a protein graph $G^{p}$. Then the drug graph $G^{d}$ and the protein graph $G^{p}$ can be fused into a hybrid graph $G^{h}$ as follows:


(6) $$G^{H}=\left[\begin{array}{cc} G^{p} & 0\\ 0 & G^{d} \end{array}\right]$$

However, proteins and drugs are still isolated and cannot interact in $G^{h}$. Therefore, we design a special central node to connect the nodes of $G^{d}$ and $G^{p}$ as a bridge for information exchange, as shown in the lower part of Fig. 2. In this way, the updated mixed graph $G^{H^{\prime }}$ is expressed as:


(7) $$G^{H^{\prime }}=\left[\begin{array}{ccc} G^{p} & 0 & 1\\ 0 & G^{d} & 1\\ 1 & 1 & 1 \end{array}\right]$$

To enable message passing in the graph structure, each node needs a feature vector to represent itself. We obtain drug and protein embeddings through a sequence-based feature generator with BiLSTM outputs.

There are no specific embeddings corresponding to the features of the central node. According to our design, this central node should be able to characterize the overall properties of drugs and proteins. Therefore, we choose the embeddings specially marked “[CLS]” at the beginning of the SMILES and sequence embedding as the features of the central node. It is designed to capture the global representation of the input sequence. Since the features of the drug $h_{d}$ and the protein $h_{p}$ in the feature generator have the same dimension, we can directly use the average of two “[CLS]” embeddings as the feature of the central node. Thus, the central node may have features that can represent the whole hybrid graph.

Then, we use multilayer GIN for the message passing of the hybrid graph as follows:


(8) $$O_{g}=\mathrm{GIN}(G^{H^{\prime }}).$$

GIN is based on the Weifeiler-Lehman test. Due to injective aggregation, GIN approximates the maximum discriminative power of GNN, described as follows: where hθ is an injective function such as a multilayer perceptron, ϵ is an artificial hyperparameter, and *Ni* is the set of neighborhood nodes of node *i*.


(9) $$h_{v}^{k}=h\theta ((1+\epsilon )h_{v}^{k-1}+\sum_{j\in Ni}h_{j}^{k-1}),$$

### 3.3 Adaptive feature training

We also propose an adaptive feature training strategy to overcome the difficulty of GNN in expressing conformational changes of proteins and ligands during the interaction. This strategy adjusts node features to ensure that each node obtains neighborhood information. Meanwhile, we introduces an adaptive feature generation mechanism to fit optimal features and balance the contributions of the feature generator and HGNN. The detailed design is described in the Supplementary Section S1.1.

### 3.4 SMILES tokenizer

Previous methods for predicting DTI used SMILES as input, but standard word segmentation methods can lead to the over-segmentation of atoms, destroying the molecule’s information. To address this, an atomic-level tokenizer is designed using RDKit to get all-atom categories in the dataset and record their positions in each SMILES. This allows for the extraction of molecule features corresponding to embedding features from the drug features obtained from BiLSTM. Node features of each atomic node are obtained for HGNN. This ensures that each atom in the drug has a one-to-one node feature by the feature generator. The detailed design is described in the Supplementary Section S1.2.

## 4 Experiment

### 4.1 Training setting

Our experiments split the training, validation, and test sets in a ratio of 8:1:1. Our data splitting was based on the TDC library (Huang *et al.* 2022). For the random setting, the Davis dataset was split into 24 044, 3006, and 3006 samples for the training, validation, and test sets, respectively, while the KIBA dataset was split into 94 467, 11 808, and 11 808 samples. For the cold drug setting, the Davis dataset was partitioned into 54, 7, and 7 non-overlapping drugs for the training, validation, and test sets, respectively. Similarly, the KIBA dataset was partitioned into 1654, 207, and 207 non-overlapping drugs. Under the cold target setting, the Davis dataset was split into 354, 44, and 44 non-overlapping proteins for the training, validation, and test sets, respectively, while the KIBA dataset was split into 182, 23, and 23 non-overlapping proteins. We repeat all experiments five times by choosing different random seeds and reporting the average results.

The detailed training processes and hyper parameters setting are released in Supplementary Section S1.3.

### 4.2 Dataset and metrics

Our experiments use two well-known benchmark datasets in the DPI literature: Davis and KIBA. In the experiments, the evaluation metrics are mean squared error (MSE), Pearson correlation coefficient, Spearman’s rank correlation coefficient, concordance index (CI) (Steck *et al.* 2007), and mean reversion ($r_{m}^{2}$). The detailed calculation formulas for these metrics and datasets are released in Supplementary Section S1.4.

## 5 Result

### 5.1 Comparison experiment

We first compare with previous SOTA methods for DTA prediction on Davis and KIBA datasets. Table 1 shows the performance comparison between NHGNN-DTA and previous SOTA methods on Davis dataset. NHGNN-DTA shows the superiority of the SOTA method on all evaluation metrics, obtaining an absolute improvement of 0.06 compared to the SOTA method on the MSE, which means that the predicted MSE on the Davis dataset drops below 0.2 for the first time. The results show that the use of feature generators to implicitly obtain node features and the use of hybrid graphs to realize the information interaction between drugs and proteins at the graph level can effectively improve the accuracy of DTA prediction. The evaluation results on the KIBA dataset show the same good performance in Table 2. The result on KIBA is 0.04 (3% relative improvement) better than the SOTA method on MSE. In the Davis dataset, NHGNN outperformed the SOTA model in MSE with statistical significance (Student’s *t*-test, *P* < .05). However, the CI and Rm of NHGNN did not exhibit a significant improvement over the SOTA model (*P* > .05). In the KIBA dataset, NHGNN demonstrated superior performance over the SOTA model in both MSE and the $r_{m}^{2}$ with statistical significance (*P* < .05).

**Table 1.** The performance comparison between NHGNN-DTA and other SOTA models on the Davis dataset.^a^

Method	MSE ↓	CI ↑	$r_{m}^{2}↑$
DeepDTA	0.261(0.007)	0.878(0.002)	0.63(0.015)
MT-DTI	0.245	0.887	0.665
GraphDTA	0.229(0.005)	0.893(0.002)	0.685(0.016)
GEFA	0.228	0.893	
rzMLP	0.205	0.896	0.709
EnsembleDLM	0.202(0.005)	0.907(0.004)	
FusionDTA	0.208(0.002)	0.913(0.001)	0.743(0.002)
MgraphDTA	0.207(0.001)	0.900(0.004)	0.710(0.005)
NHGNN(Ours)	**0.196(0.004)**	**0.914(0.002)**	**0.744(0.003)**

a Bold corresponds to the best performance for each metric, and underline indicates the second best. ↑/↓ indicates that the larger/smaller the metrics, the better the model performance.

**Table 2.** The performance comparison between NHGNN-DTA and other SOTA models on the KIBA dataset.

Method	MSE ↓	CI ↑	$r_{m}^{2}↑$
DeepDTA	0.194(0.008)	0.863(0.005)	0.673(0.019)
MT-DTI	0.152	0.882	0.738
GraphDTA	0.139(0.008)	0.891(0.001)	0.725(0.018)
rzMLP	0.142	0.89	0.748
EnsembleDLM	0.138(0.003)	0.895(0.001)	
FusionDTA	0.130(0.002)	0.906(0.001)	0.793(0.002)
MgraphDTA	0.128(0.001)	0.902(0.001)	0.801(0.001)
NHGNN(Ours)	**0.124(0.002)**	**0.907(0.001)**	**0.807(0.002)**

Bold corresponds to the best performance for each metric, and underline indicates the second best.

### 5.2 Performance evaluation on more realistic settings

Previous experiments used usually random splits to divide the training, validation, and test sets. However, a random split setting may lead to overly optimistic results, as it can cause drug and protein information to leak into the test set (Mayr *et al.* 2018). For drug discovery purposes, models need to extrapolate to unseen drugs, unseen proteins, and unseen DP pairs. Therefore, in the cold-start scenario, we evaluate the performance of the DTA model using three new split methods: cold-start splitting for drugs, cold-start splitting for proteins, and for both drugs and proteins. Taking the cold start split for drugs as an example, we divide different drugs into training set, validation set and test set. That is, the drugs in the test set samples will not appear in the training set and validation set, and the training set and validation set are also different. The cold start for proteins is divided according to different proteins. These three division methods can better demonstrate the generalization of the DTA model and meet the actual situation of the DTA model in the process of new drug discovery. We compare the performance of NHGNN-DTA and previous SOTA methods on the Davis and KIBA datasets in cold-start scenarios for drugs and proteins. For a fair comparison, the splits for all methods are consistent. Experimental results for cold start scenarios are shown in Tables 3 and 4.

**Table 3.** Performance evaluation on more realistic settings of Davis datasets.

Scenario	Method	MSE ↓	CI ↑	$r_{m}^{2}↑$
Cold drug	GraphDTA	0.920(0.029)	0.678(0.036)	0.160(0.019)
	GEFA	0.847(0.012)	0.709(0.028)	0.182(0.015)
	FusionDTA	0.581(0.094)	0.737(0.012)	0.187(0.034)
	MgraphDTA	0.563(0.065)	0.729(0.022)	0.192(0.021)
	NHGNN(Ours)	**0.554(0.091)**	**0.752(0.017)**	**0.207(0.030)**
Cold target	GraphDTA	0.510(0.086)	0.729(0.012)	0.154(0.014)
	GEFA	0.433(0.022)	0.759(0.009)	0.289(0.016)
	FusionDTA	0.364(0.021)	0.826(0.011)	0.435(0.023)
	MgraphDTA	0.359(0.023)	0.813(0.008)	0.425(0.028)
	NHGNN(Ours)	**0.344(0.029)**	**0.855(0.016)**	**0.479(0.021)**
All cold	GraphDTA	0.968(0.096)	0.579(0.017)	0.026(0.016)
	GEFA	0.944(0.092)	0.610(0.029)	0.032(0.022)
	FusionDTA	0.876(0.091)	0.645(0.043)	0.072(0.048)
	MgraphDTA	0.874(0.090)	0.636(0.021)	0.071(0.041)
	NHGNN(Ours)	**0.857(0.096)**	**0.665(0.038)**	**0.087(0.051)**

Bold corresponds to the best performance for each metric, and underline indicates the second best.

**Table 4.** Performance evaluation on more realistic settings of KIBA datasets.

Scenario	Method	MSE ↓	CI ↑	$r_{m}^{2}↑$
Cold drug	GraphDTA	0.471 (0.047)	0.713(0.002)	0.342(0.007)
	GEFA	0.464(0.032)	0.721(0.003)	0.346(0.006)
	FusionDTA	0.429(0.031)	0.748(0.005)	0.364(0.012)
	MgraphDTA	0.425(0.047)	0.746(0.002)	0.366(0.016)
	NHGNN(Ours)	**0.385(0.029)**	**0.756(0.007)**	**0.400(0.015)**
Cold target	GraphDTA	0.469(0.089)	0.610(0.035)	0.368(0.057)
	GEFA	0.462(0.091)	0.636(0.037)	0.362(0.052)
	FusionDTA	0.439(0.062)	0.685(0.032)	0.390(0.067)
	MgraphDTA	0.435(0.055)	0.674(0.028)	0.382(0.047)
	NHGNN(Ours)	**0.382(0.071)**	**0.732(0.041)**	**0.452(0.054)**
All cold	GraphDTA	0.676(0.113)	0.601(0.030)	0.149(0.067)
	GEFA	0.639(0.065)	0.628(0.047)	0.152(0.035)
	FusionDTA	0.587(0.086)	0.641(0.023)	0.193(0.053)
	MgraphDTA	0.590(0.094)	0.626(0.028)	0.182(0.012)
	NHGNN(Ours)	**0.565(0.094)**	**0.649(0.037)**	**0.218(0.047)**

Bold corresponds to the best performance for each metric, and underline indicates the second best.

In the Davis dataset, our model improved metrics by an average of 4%, 6%, and 8% for the three cold start settings. On the KIBA dataset, the average improvement was 7%, 12%, and 8%. NHGNN exhibited a statistically significant improvement over the SOTA model in all metrics under three cold start scenarios in both the Davis and KIBA datasets (*P* < .05). Compared with previous methods, we specifically use a feature generator to obtain high-dimensional features of drug and protein nodes instead of manually setting atomic and protein features, which may be the reason for the stronger generalization of NHGNN-DTA. The features obtained by the feature generator can better characterize drugs or proteins that were not encountered during training.

### 5.3 Ablation study

We did ablation experiments on NHGNN-DTA’s components to see which ones contribute the most to its predictive ability. We tested the feature generator, no feature pretraining, no feature updating, and the full model on the Davis dataset, and found that feature updating was the most important for HGNN. Additionally, the complete NHGNN-DTA had the best performance on all metrics, showing the importance and effectiveness of all components and methods. Results are in the Supplementary Section S2 and Supplementary Table S2.

## 6 Interpretability analysis

Since NHGNN contains an attention mechanism, we can analyze the attention weights of the final output of the feature generator $O_{\text{con}}$. Since its upstream input contains the feature of drug $h_{d}$ and protein $h_{p}$, its attention weights may demonstrate the interaction of DP pairs. Therefore, we visualize the central node’s attention weights to represent the contribution of each amino acid and atom to the final affinity prediction.

The interpretability of NHGNN-DTA can be demonstrated by the weight analysis of attention. The visualization of the model’s weights can further explain the DTA predictions, thereby helping to understand the underlying mechanisms of drug discovery based on proteins as targets. As cases for weight visualization, we choose 1OUK and 4XUF in the Protein Data Bank (PDB) database (Rose *et al.* 2016), i.e. the crystal structures obtained from *in vitro* experiments.

**Figure 3.**
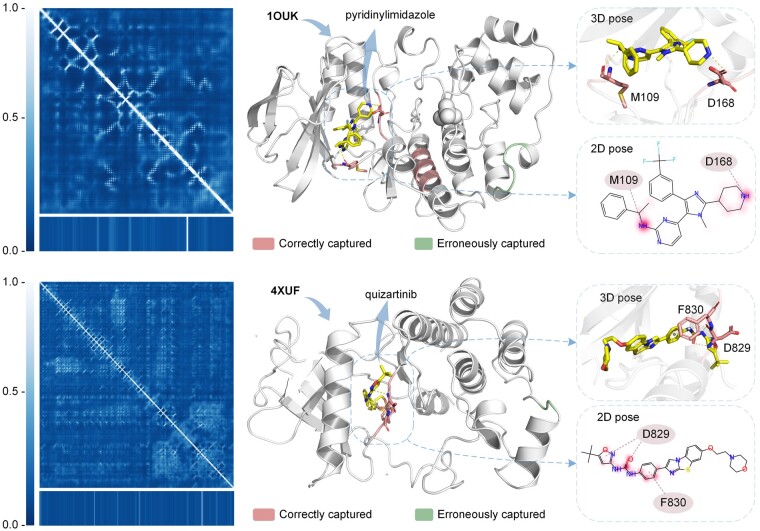
Visualization of attention weights for DP pairs. The upper part represents the MAP kinase p38 alpha and its binding ligand pyridinylimidazole inhibitor (PDB ID: 1OUK), and the lower part represents the protein FMS-like tyrosine kinase 3 protein and its binding ligand quizartinib inhibitor (PDB ID: 4XUF). In the figure, the left is the contact map, where the bottom is the corresponding attention value. On the right are the crystal structure, 2D pose, and 3D pose of the protein and its ligand-bound state. In the crystal structure, the top 20 residues of the attention weight are highlighted in red and cyan, with red indicating the correct region captured by the model and cyan indicating the wrong region. 3D pose and 2D pose show confirmed interactions and interacted residues. The 2D pose also shows the attention weights of the drug highlighted in red, with shades of color representing the magnitude of the weights.

Figure 3 shows the visualization of attention weights for DP pairs. 1OUK is shown in the upper part of the Fig. 3, where the protein contact map and corresponding weights are shown on the left. In the crystal structure, the top 20 residues of the attention weight are highlighted in red and cyan on the right, where red is the region of correctly captured residues by NHGNN-DTA and cyan is the region of the wrongly captured residue. The L108-G110 and L167-G170 sites of the protein are in high interest positions, and this number falls in the docking pocket. In the 3D pose, the confirmed interaction residues M109 and D168 received higher weights. Also, the L129-138L position falls right into another potential pocket. In addition, the model also erroneously captured the residue region V273-277A, which is not in the docking pocket. In the 2D pose, the substructure attention value of the drug is highlighted in red. Meanwhile, the attentional weights of drugs are mainly focused on atoms 2 and 25, corresponding to the key atom N that forms interactions with proteins.

The bottom half of Fig. 3 shows the crystal structure of 4XUF, and the protein attention weight was highest at C828-G831, which is in the docking pocket. However, non-docking pocket regions of G905 and F906 were incorrectly captured. The model correctly captured key residues F830 and D829 that interact with ligands. The drug weight was mainly on O and benzene rings, which correspond to the interacting protein residues. Although the model miscaptured some pockets, NHGNN-DTA can still focus on most docking sites and related drug substructures, indicating its interpretability and ability to explore potential DTAs.

## 7 Case study

**Figure 4.**
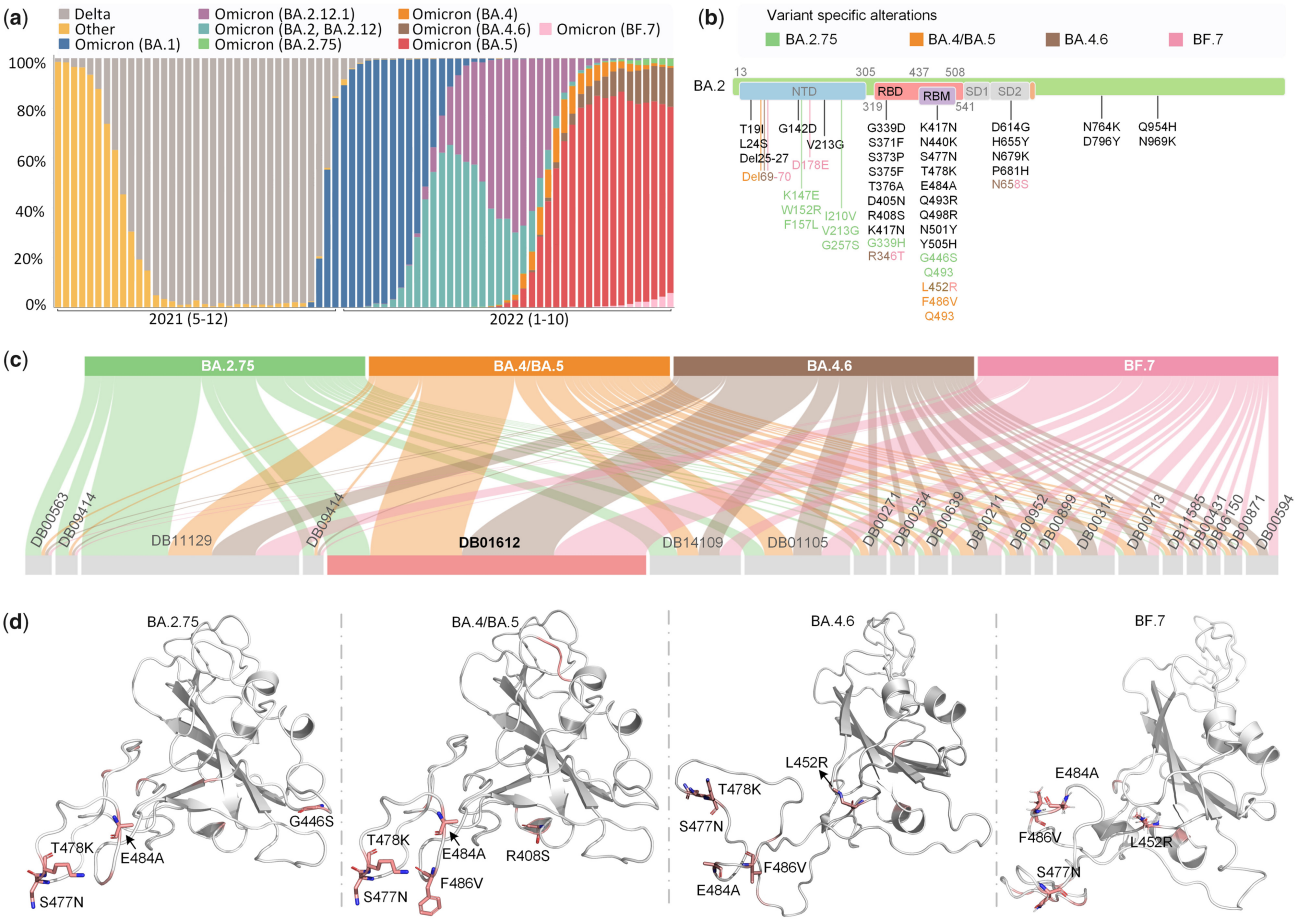
Flowchart and results of the NHGNN-DTA case study applied to drug repurposing screening of Omicron variants against SARS-CoV-2. (a) Distribution of variants sequence and reported by the Centers for Disease Control and Prevention National SARS-CoV-2 Strain Surveillance program. It shows the type of epidemic variants from May 2021 to October 2022, with time on the abscissa and percentage of variants on the ordinate. (b) SARS-CoV-2 Omicron BA.2.75, BA.4/BA.5, BA.4.6, and BF.7 mutation sites relative to BA.2. The color indicates the corresponding variant contains the mutation. (c) Sankey diagram of predicted affinity of 5 targets with top 20 FDA-approved drugs. The width represents the magnitude of the predicted affinity. (d) Visualization of the attention weights of the model when the RBD of 5 variants and Amyl Nitrite are used as the input of the model. The top 10 model output attention sites are indicated in red, where the sites shown in stick form are the mutation sites of Omicron variant.

To further verify the effectiveness of NHGNN-DTA, we applied this model to the repurposing of antiviral drugs of the currently circulating SARS-CoV-2 Omicron variants. Figure 4a illustrates the monitoring of SARS-CoV-2 sample source variants by the Centers for Disease Control and Prevention. The proportion of variants of COVID-19 in cases is changing constantly. As of October 8, 2022, most cases belong to five prevailing variants: SARS-CoV-2 Omicron BA.2.75, BA.4/BA.5, BA.4.6, and BF.7. The above five variants were all derived from the mutation of Omicron BA.2. Therefore, we analyzed the mutation sites of the five variants relative to BA.2, as shown in Fig. 4b, we find that most of the mutation sites are located in the receptor binding domain (RBD) and its internal receptor binding motif (RBM) region.

Therefore, we selected the RBDs of the above five variants as targets. The crystal structures of Omicron BA.2.75 and BA.4/BA.5 were obtained from the PDB database, while the crystal structures of Omicron BA.4.6 and BF.7 have not been Publicly available, so modeled by trRosetta server (Du *et al.* 2021). Both structures were built with restraints from both DL and homologous templates. Besides, both of the results confidence of the model is high. The DrugBank database (Wishart *et al.* 2018) was used to obtain 2509 FDA-approved drugs as potential inhibitors waiting for screening. Then, we used DP pairs as the input of the model. Through NHGNN-DTA, we predicted the affinities of those drugs to five variants and then selected the drugs with the top 20 predictive affinities. The maximum predicted affinity was obtained between Amyl Nitrite (id: DB01612) and the five variants, as shown in Fig. 4c. The model predicted the affinity of Amyl Nitrite for BA.2.75, BA.4/BA.5, BA.4.6, and BF.7 to be 0.153, 0.071, 0.073, and 0.073 nM, respectively. The predicted results suggest that Amyl Nitrite may be a strong inhibitor of Omicron and is more effective for the new variants.

Using NHGNN-DTA, we analyzed the mechanism of AmylNitrite and targets by outputting attention weights of five target variants. As shown in Fig. 4d NHGNN-DTA captured two key mutation sites, S477N and E484A, for all targets, indicating Amyl Nitrate’s potential mechanism on Omicron variants. The model also successfully identified other key mutation sites for the variants, such as G446S, F486V, and L452R, while capturing some sites not belonging to the variant, such as T478K in BA.2.75, possibly due to similar fragments between variants. Overall, the case study demonstrated NHGNN-DTA’s capability to identify target mutation sites and strong interpretability.

Our selected Amyl Nitrate seems to have a relatively good affinity for the recently circulating omicron variant of SARS-CoV-2, and interacts with key mutations of RBM in the RBD of the Omicron variants. However, in the article, we aim to develop an efficient computational method for drug repurposing, so the screened drugs do not represent the actual efficacy. Specific drug effects require further *in vitro* assays, *in vivo* assays, and clinical trials.

## 8 Conclusion

In this article, we propose an HGNN that can adaptively generate features. Unlike sequence-based methods, we exploit drug and protein structures by constructing hybrid graphs containing amino acid and atomic nodes. Different from graph-based methods, this model realizes the adaptive update of node features by building a feature generator to increase the information interaction between drugs and proteins at the graph level. In general, NHGNN-DTA overcomes the problems that sequence-based methods cannot obtain graph structure information and graph-based methods cannot construct suitable node features. It also combines the advantages of the above two methods. This is the first time the integration of two DTA prediction methods has been achieved according to the previous literature review. Extensive experiments show that NHGNN-DTA achieves significant improvements on two benchmark datasets compared to recent SOTA methods. In the scenario of cold start for drugs and proteins, NHGNN-DTA demonstrated its stronger generalization and robustness. Through attention visualization and case study, we demonstrate the substructure information capture ability of the model. The case study implies that Amyl Nitrite may be a potentially effective drug candidate against SARS-CoV-2 Omicron variants. Future work will focus on generating potential DP pairs to accelerate the process of drug discovery.

## Supplementary Material

btad355_Supplementary_Data

## Data Availability

The data underlying this article are available in https://github.com/hehh77/NHGNN-DTA.
