# Supplementary material for: NHGNN-DTA: a node-adaptive hybrid graph neural network for interpretable drug–target binding affinity prediction
Source: Bioinformatics. 2023 May 30;39(6):btad355. doi: 10.1093/bioinformatics/btad355 (PMC10287904; doi:10.1093/bioinformatics/btad355)
Supplement: btad355_Supplementary_Data [file btad355_supplementary_data.pdf]

Supplementary Materials for ‘NHGNN-DTA: A Node-adaptive Hybrid Graph Neural Network for Interpretable Drug-target Binding Affinity Prediction’

Haohuai He†[a], Guanxing Chen†[a], and Calvin Yu-Chian Chen *[a,b,c]

[a]Artificial Intelligence Medical Research Center, School of Intelligent Systems Engineering, Sun Yat-sen University, 510275, China

[b]Department of Medical Research, China Medical University Hospital, Taichung 40447, Taiwan

[c]Department of Bioinformatics and Medical Engineering, Asia University, Taichung 41354, Taiwan

This PDF file includes:

- Supplementary Methods and Materials
- Supplementary Results
- Supplementary Tables S1-S2
- Supplementary Figures S1

*Corresponding author: chenyuchian@mail.sysu.edu.cn

†These authors contributed equally to this work.

Supplementary materials

1 Methods and Materials

1.1 Adaptive Feature Training

The hybrid graph $G^{H'}$ connected through the central node is static and GNN transmits messages through neighborhood messaging. Therefore, due to the sparse structure of proteins and drug molecules, it is difficult for GNN to express the conformational changes of proteins and ligands during the interaction by messages passing. To address this issue, we incorporate an adaptive feature training strategy. Adaptive feature training can dynamically adjust the features of nodes to ensure that each node obtains the neighborhood information. Therefore, through multiple node feature adjustments, NHGNN may overcome the above problems.

The input to the HGNN model depends on the output of the feature generator. Therefore, we design models to adaptively generate features that best characterize amino acid and atomic nodes. To achieve this goal, we innovatively construct an adaptive feature generation mechanism to fit optimal features. Specifically, we jointly train the pre-trained feature generator and HGNN in the training phase. We set a hyperparameter θ and get the final output as follows:

$$O = O_g * \theta + O_{pre} * (1 - \theta). \tag{1}$$

The role of the hyperparameter θ is to balance the contributions of the feature generator and HGNN. Through this joint training, the feature generator can update the node features again according to the back-propagation of the GNN during the training process, so that the node features become better features of the GNN. During the training of the model, there will be some problems with constantly updating the node features. First, features drift, meaning that the mean and variance of features change. Also, changing the samples at every epoch makes the model non-convergence. Therefore, we adopt two methods to solve the above problems.

Inspired by [5], we add a LayerNorm layer after the BiLSTM layer to ensure that the distribution of features will not have significant change. In addition, we set the feature update interval frequency to multiple epochs to ensure that the model can converge faster and maintain adaptive learning of amino acid and atomic node features.

1.2 SMILES Tokenizer

In previous sequence-based DTA prediction methods, the SMILES of drugs are passed into the tokenizer as a complete input, and the acquired token is based on the natural language processing word segmentation methods, e.g., n-gram. However, SMILES may have atoms that should not be split according to frequency and other attributes like ordinary texts. For example, ‘Cl’ should be the notation for a chlorine atom, not divided into ‘C’ and ‘l’. Therefore, the use of splitting methods in previous work leads to excessive word segmentation by the tokenizer, which may destroy the complete information of the drug molecules contained in SMILES. To this end, we design a special atomic-level tokenizer to ensure thatwe can get atomic one-to-one correspondence. First, we use the RDKit tool to get all atom categories present in the dataset, then add them to the vocabulary, and remove the atom’s subcategories for further phasing. After reading all SMILES, the position of each atom P_i in the molecule is obtained. By recording the P_i of each SMILES in the dataset, we extract the molecule features corresponding to the embedding features from the drug features h_d obtained from the BiLSTM in the adaptive feature generator as follows:

$$G_d = h_d[p_0, p_1, \cdots, p_i, \cdots, p_N], \tag{2}$$

where N is the atom number of the drug. Finally, we obtain the node features of each atomic node in the G_d of HGNN. Through the above design,, we ensure that each atom in the drug can get one-to-one node features by feature generator.

1.3 Training Setting

To make the feature vectors come from an adaptive feature generator that can better characterize nodes, we first pre-train the feature generator for 200 epochs, and then simultaneously train the feature extractor and GNN through joint training. We use a feature generator to update the node features of the hybrid graph every 40 epochs.

We used Adam optimizer to optimize parameters, and used a cosine annealing learning rate (lr) adjustment strategy. As shown in the following formula:

$$lr = lr_{min} + \frac{1}{2}(lr_{max} - lr_{min})(1 + \cos(\frac{T_{cur}}{T_{max}}\pi)), \tag{3}$$

where lr_{min} and lr_{max} are the upper and lower limits of lr , and T_{max} is the period in which lr fluctuates according to the cosine.

In the training stage, we used MSE loss function to calculate the loss. Every 40 epochs, the feature generator will update the feature representation of the nodes in the hybrid graph. In addition, we reduced the learning rate of the feature generator model in the training stage to prevent it from changing greatly, since it has learned the feature representation of DPI prediction in the pre-training stage. We tried {5e-4, 1e-3, 2e-3, 5e-3} on LR. For embedding dimension, we tried {64,128,256}. For GIN input dimension, we tried {64,128,256}. For hidden dimension, we tried {64,128,256}. We use the training set in the data set for multiple random attempts, and select the best performance in the validation set as the final parameter setting. All hyper parameters are shown in Table S1. All experiments were run on Linux OS with a NVIDIA GeForce RTX A6000 GPU and a processor Intel(R) Xeon(R) Silver 4210R CPU @ 2.40GHz.

1.4 Datasets and Metrics

1.4.1 Davis

Davis et al. [1] tested 72 kinase inhibitors for interaction with 442 kinases, covering more than 80% of the human catalytic protein kinome. The dataset contains 30,056 DP pairs, each of which includes the SMILES of the drug and the sequence of the protein, and the IC_{50} activity value as a label. We convert the activity value to negative logarithm pIC_{50}.

1.4.2 KIBA

To exploit the complementary information captured by various bioactivity types, including IC50, K_i, and K_d, Tang et al. [4] introduced a model-based ensemble approach, termed KIBA, to generate an integrated drug-target bioactivity matrix. It contains 118,083 DP pairs, consisting of 2,068 drugs and 229 proteins. Each set of data contains a label that uses the KIBA score as the binding activity value.

1.4.3 Mean square error (MSE)

MSE is a measure that direct evaluate the error, it defined as follows:

$$MSE = \frac{1}{N}\sum_{N}^{i=1}(Y_i - Y_n^{'})^2, \tag{4}$$

where N is the number of samples, Y_i represents the predicted value of the model for the ith sample, $Y_N^{'}$ represents the label of the ith sample.

1.4.4 Pearson

Pearson measures the linear correlation between the predicted value p and the label y, it defined as follows:

$$Pearson = \frac{\phi(p, y)}{\phi(p)\phi(y)}, \tag{5}$$

where $\phi(p, y)$ is the covariance between the predicted value and the label, $\phi(p)$ is the standard deviation of p, and $\phi(y)$ is the standard deviation of y.

1.4.5 Spearman

Spearman is a metric to measure the rank correlation, it defined as follows:

$$Spearman = 1 - \frac{6\sum_{i=1}^{n} d_i^2}{n(n^2 - 1)}, \tag{6}$$

where d_i is the difference between two ranks in the predicted values and labels.

1.4.6 Concordance index (CI)

CI is a metric that quantifies ranking quality and the predictive accuracy of the model. CI is defined as follows:

$$CI = \frac{1}{Z}\sum_{\delta_j > \delta_i} h(b_i - b_j), \tag{7}$$

where b_i is the prediction for δ_i, b_j is the prediction for δ_j, Z is a normalization constant, and $h(x)$ is the step function. The step equation $h(x)$ is defined as follows:

$$h(x) = \begin{cases} 1 & \text{if } x > 0 \\ 0.5 & \text{if } x = 0 \\ 0 & \text{if } x < 0 \end{cases} \cdot \tag{8}$$

1.4.7 Mean reversion (r_m^2)

r_m^2 is a measure to evaluate the external prediction performance of the model, which is defined as follows:

$$r_m^2 = (1 - \sqrt{(r^2 - r_0^2)}) * r^2, \tag{9}$$

where r is the squared correlation coefficient with intercept and r_0 is the coefficient without intercept.

2 Results

Figure S1 shows the performance of NHGNN-DTA and other methods more intuitively. The three subplots in the upper part represent the performance of methods on the Davis dataset. The left pie chart shows the comparison of MSE, the middle pie chart shows the comparison of CI, and the right is a scatter plot of the experimental affinity and NHGNN-DTA predicted affinity of samples on the test set. The three subgraphs in the lower part of Figure S1 are the results of the KIBA dataset. Pie charts show the advantages of NHGNN-DTA more intuitively. And through the scatter plot, we can visualize that most of the results predicted by the model and the real value are close to a straight line with a slope of 1, which represents a very accurate prediction of the model. In addition, for better visualization of the results, we removed two outliers from the KIBA test set, whose real and predicted values are

2.1 Ablation Experiment

Additionally, we conducted ablation experiments to investigate the predictive ability gain of different components of NHGNN-DTA. The results of the ablation experiments performed on the Davis dataset are shown in Table S2. We tested the performance of only the feature generator, no feature pretraining, no feature update, and the full model. The experimental results show that the performance of NHGNN-DTA degrades the most without feature updating, which indicates the importance of feature updating for HGNN. Furthermore, the complete NHGNN-DTA achieves the best results on all metrics, which demonstrates the necessity and effectiveness of each component and method of NHGNN-DTA.

Table S1: Hyper parameters setting.

	Hyperparameter	Value
Pre-training	Lr	1e-3
	Number of epochs	200
	Mini-batch size	128
	Lr_{min}	5e-4
	Period	20
	Embedding dimension	256
	BiLSTM dimension	128
	Number of Heads	8
	Dropout rate	0.2
	Num of BiLSTM layers	2
Training	Feature generator Lr	1e-4
	GNN Lr	1e-3
	Number of epochs	400
	Mini-batch size	128
	Period	20
	Lr_{min}	2e-4
	GIN input dimension	256
	Hidden dimension	128
	Dropout rate	0.2

Table S2: Results of ablation experiments on Davis data set. 'Wo HGNN' means using only feature generator for DTA prediction, 'Wo pre-training' means directly training the entire model without pre-training the feature generator, 'Wo feature update' means not updating node features, 'full' means using the complete model, NHGNN-DTA.

	MSE ↓	CI ↑	r_m^2↑
Wo HGNN	0.210	0.901	0.733
Wo pre-training	0.213	0.902	0.733
Wo feature update	0.220	0.897	0.715
Full	**0.196**	**0.914**	**0.744**

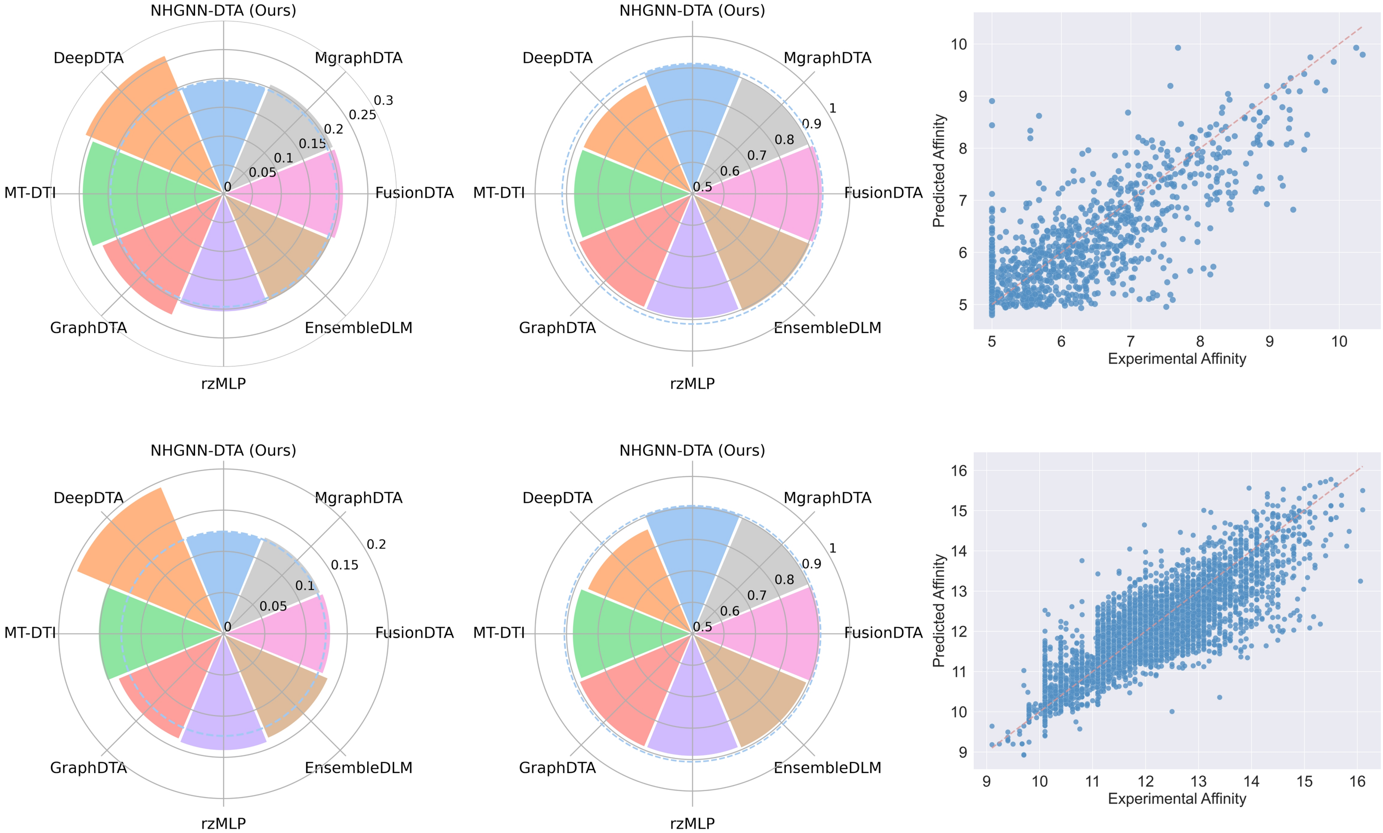

Figure S1. Test results of NHGNN-DTA and other DTA methods on Davis and KIBA datasets. The three subplots in the upper part represent the performance of methods on the Davis dataset, while the three subplots in the lower part represent the performance of methods on the KIBA dataset. The left pie charts show the comparison of MSE, the middle pie charts show the comparison of CI, and the right is the scatter plots of the experimental affinity and NHGNN-DTA predicted affinity of samples on the test set.

References

[1] M. I. Davis, J. P. Hunt, S. Herrgard, P. Ciceri, L. M. Wodicka, G. Pallares, M. Hocker, D. K. Treiber, and P. P. Zarrinkar. Comprehensive analysis of kinase inhibitor selectivity. *Nature biotechnology*, 29(11):1046–1051, 2011.

[2] H. He, G. Chen, and C. Yu-Chian Chen. 3dgt-ddi: 3d graph and text based neural network for drug–drug interaction prediction. *Briefings in Bioinformatics*, 23(3):bbac134, 2022.

[3] Y. Liu, L. Wang, M. Liu, X. Zhang, B. Oztekin, and S. Ji. Spherical message passing for 3d graph networks. *arXiv preprint arXiv:2102.05013*, 2021.

[4] J. Tang, A. Szwajda, S. Shakyawar, T. Xu, P. Hintsanen, K. Wennerberg, and T. Aittokallio. Making sense of large-scale kinase inhibitor bioactivity data sets: a comparative and integrative analysis. *Journal of Chemical Information and Modeling*, 54(3):735–743, 2014.

[5] A. Vaswani, N. Shazeer, N. Parmar, J. Uszkoreit, L. Jones, A. N. Gomez, Ł. Kaiser, and I. Polosukhin. Attention is all you need. *Advances in neural information processing systems*, 30, 2017.
